# A Gateway between Recent and Remote Memory

**DOI:** 10.3389/fnins.2012.00153

**Published:** 2012-10-09

**Authors:** Gisella Vetere, Martine Ammassari-Teule

**Affiliations:** ^1^Istituto di Biologia Cellulare e Neurobiologia, Consiglio Nazionale delle Ricerche (CNR)Rome, Italy; ^2^Santa Lucia FoundationRome, Italy

Maintaining memories over time requires a highly dynamic process of brain activation based on time-dependent recruitment of subcortical and cortical regions (Frankland and Bontempi, 2005; Squire and Bayley, 2007). The necessity for a such complex network is due to the fact that, as time elapses, memories tend to stabilize but also to preserve only partial aspects of the original event. Stabilization, generalization, and updating of remote memory therefore require that the involvement of single regions, as well as the dialog between regions, fluctuates over time.

Much is known about the predominant role of the hippocampus (HPC) in the formation and consolidation of recent memories. For example, lesioning this region immediately after acquisition impairs memory recalled shortly after training but has no effect when the recall takes place long after (Anagnostaras et al., 1999). In support to this, neuronal activity (Bontempi et al., 1999; Frankland et al., 2004) and connectivity (Restivo et al., 2009) are selectively enhanced in the HPC at short post-training intervals. However, the role of the HPC in memory storage is time-limited (Scoville and Milner, 1957; Squire, 1992). Evidence has accumulated that long after a memory is formed, the HPC is disengaged in favor of medial prefrontal cortex (mPFC) regions including the anterior cingulate (Restivo et al., 2009), the infralimbic (Vetere et al., 2011b) and the orbitofrontal cortices (Lesburgueres et al., 2011). Neuronal networks in these regions are, in fact, selectively remodeled at remote time-points (Restivo et al., 2009). Moreover, the persistence of these lately occurring structural changes is necessary for the persistence of memory (Vetere et al., 2011a). Surprisingly, in contrast with the intuitive idea that the neocortex might exclusively be dedicated to the storage of remote memory traces, it has been recently shown that lesions to lateral entorhinal cortex disrupt both recent and remote memory (Morrissey et al., 2012). Because this region is intimately connected with the HPC and the mPFC (Jones and Witter, 2007; Kerr et al., 2007), it therefore provides a crucial anatomical link potentially involved in the transfer of information between the two regions.

There is evidence that, during a training episode, HPC activity is dominated by theta oscillations. Immediately after, HPC neurons burst synchronously constituting sharp waves which can propagate to other structures. Indeed, mPFC neurons are active during the recall of remote memories and their neuronal firing became selective for the association formed during the learning episode (Takehara-Nishiuchi and McNaughton, 2008). Nevertheless, how these regions interact to stabilize a recently acquired information into a long lasting memory was still unclear.

In their paper published last year in Frontiers in Behavioral Neuroscience, Takehara-Nishiuchi et al. (2012) highlight this question by analyzing theta synchronization between LEC and HPC, and between LEC and the mPFC, their idea being that initial encoding of memories might be supported by LEC-HPC synchronization, and remote memory recall by LEC-mPFC synchronization. According to this view, the LEC might be initially involved in (i) transmitting sensory information to the HPC where a memory trace would be formed and (ii) recruiting the mPFC for future stabilization of the trace in the neocortex. Their prediction was, therefore, that theta synchronization between LEC and HPC, and between LEC and mPFC should be enhanced already during training. However, only the enhancement of theta synchronization between LEC and mPFC should persist over time to ensure long term encoding of the trace in neocortical networks.

To this aim, they trained two different groups of rats in a trace eyeblink conditioning consisting in the presentation of a neutral stimulus (CS) followed by a mild electric shock to the eyelid (US). The first group was trained for 10 days while the second group was trained and tested 1 month later. Local field potentials (LFPs) in the HPC, the limbic part of mPFC, and LEC during conditioning and, for the second group, during long term retention were recorded. Consistent with previous observations, a robust amplitude of theta oscillations was observed in the HPC and the mPFC (Munera et al., 2001; Paz et al., 2008; Darling et al., 2011) but also in the LEC.

Then, to detect an increase in communication between HPC, mPFC, and LEC following the CS presentation, they measured phase synchronization between theta oscillations in simultaneously recorded pairs of LFPs (Lachaux et al., 1999; Fell and Axmacher, 2011).

Recordings performed after the CS onset during the first acquisition session revealed an increase in synchronization between LEC and HPC both in rats exhibiting the conditioned eyeblink response as well as in rats which did not exhibit it, suggesting that the initial HPC-LEC dialog is not an index of conditioning. In agreement with their prediction, they then showed that during the remote retention session (i) the amplitude of HPC theta oscillations was lower than during acquisition, and that, (ii) in line with the time-limited engagement of HPC in consolidation process, theta synchronization of HPC with LEC was decreased. More intriguingly, they observed that theta amplitude in the LEC and the mPFC increased during training and became gradually higher on trials during which rats showed the conditioned response. The synchronization between the two regions then remained elevated during the remote sessions, suggesting a role for LEC-mPFC ensembles in driving the late phase of consolidation and related modifications in prefrontal neuronal firing.

These experiments, which identify the LEC as a gateway between the HPC and the mPFC, provides a remarkable contribution to the understanding of the mechanisms supporting remote memory consolidation. In particular, the segregation in different LEC layers of anatomical connections with HPC and mPFC reveals the ability of this region to elaborate sensory information through a direct dialog with hippocampal neurons, to maintain a long lasting communication with mPFC neurons, and, via intra-LEC connections, to transfer the information processed at the HPC level to mPFC circuits.
